# Comparison of different postoperative paın managements in patients submitted to transperitoneal laparoscopic renal and adrenal surgery

**DOI:** 10.1590/S1677-5538.IBJU.2013.0238

**Published:** 2015

**Authors:** Altug Tuncel, Melih Balci, Aysun Postaci, Yilmaz Aslan, Ali Atan

**Affiliations:** 1 Third Department of Urology, Ministry of Health, Ankara Numune Research and Training Hospital, Ankara, Turkey; 2 Second Department of Anaesthesiology, Ministry of Health, Ankara Numune Research and Training Hospital, Ankara, Turkey

**Keywords:** Laparoscopy, surgery [Subheading], Pain

## Abstract

**Purpose:**

We compared the effects of local levobupivacaine infiltration, intravenous paracetamol, intravenous lornoxicam treatments on postoperative analgesia in patients submitted to transperitoneal laparoscopic renal and adrenal surgery.

**Materials and Methods:**

Sixty adult patients 26 and 70 years who underwent laparoscopic renal and adrenal surgery were randomized into three groups with 20 patients each: Group 1 received local 20mL of levobupivacaine 0.25% infiltration to the trocar incisions before skin closure. In group 2, 1g paracetamol was given to the patients intravenously 30 minutes before extubation and 5g paracetamol was given intravenoulsy in the 24 postoperative period. In group 3, 8mg lornoxicam i.v. was given 30 minutes before extubation and 8mg lornoxicam i.v. was given in the 24 postoperative period. In the postoperative period, pain scores, cumulative tramadol, and additional pethidine consumption were evaluated.

**Results:**

Postoperative pain scores significantly reduced in each group (p < 0.05). Although pain levels of the groups were not significantly different at 1, 2, 4, 8, 12 and 24 hours postoperatively, cumulative tramadol consumptions were higher in group 1 than the others. (Group 1 = 370.6 ± 121.6mg, Group 2: 220.9 ± 92.5mg, Group 3 = 240.7 ± 100.4mg.) (p < 0.005). The average dose of pethidine administered was significantly lower in groups 2 and 3 compared with group 1 (Group 1: 145mg, Group 2: 100mg, Group 3: 100mg) (p = 0.024).

**Conclusions:**

Levobupivacaine treated group required significantly more intravenous tramadol when compared with paracetamol and lornoxicam groups in patients submitted to transperitoneal laparoscopic renal and adrenal surgery.

## INTRODUCTION

Since the first laparoscopic nephrectomy performed in 1991 by Clayman and associates (1), laparoscopic surgery has been applied successfully in various urological operations. Laparoscopic renal and adrenal surgeries including radical/simple nephrectomy, partial nephrectomy, nephroureterectomy, pyeloplasty, adrenalectomy, have demonstrated success rates comparable to those of open surgery, with lower postoperative morbidity including faster recovery time, shorter hospital stay, less pain, and in some cases, fewer complications (2, 3). Despite the minimally invasive nature of these operations, postoperative pain in patients undergoing laparoscopic procedures may be substantial and limit an otherwise expeditious recovery. Several postoperative pain managements with additive and synergic drugs aim to provide adequate analgesia.

The objective of the present study was to compare the efficacies of intravenous paracetamol, intravenous lornoxicam and levobupivacaine infiltration into the incisional trocar sites therapies on postoperative analgesia in patients submitted to transperitoneal laparoscopic renal and adrenal surgery.

## MATERIAL AND METHODS

This study was approved by the Institutional Review Board (Registration # 10/43) and each patient's consent for the use of their information was taken in a written form. Between September 2010 and October 2011, 60 adult ASA I-III physical status patients (32 male and 28 female) with a mean age of 48.1 years (range; 26 to 70) who underwent transperitoneal laparoscopic renal and adrenal surgery were included in this study. Of the patients in the present study, 6 patients underwent renal cyst decortication (Left: 2, Right: 4), 9 patients underwent simple nephrectomy (Left: 4, Right: 5), 12 patients underwent adrenalectomy (Left: 5, Right: 7) and 33 patients underwent radical nephrectomy (Left: 21, Right: 12). Standard transperitoneal laparoscopic procedures with a 3-trocar technique for left side and 4-trocar technique (except for renal cyst decortication) for right side was performed in all patients in a 450 semi-flank position (4). CO2 pneumoperitoneum at a maximum pressure of 14 mmHg was maintained. The intact kidney specimens were removed from the umbilical trocar site using peri-umbilical incision in patients who underwent simple and radical nephrectomy. Also, the intact adrenal specimens were removed from the working trocar sites in patients who underwent adrenalectomy. The excised cyst walls were removed via working trocar. We installed Jackson-Pratt drain at the end of all operations. All operations were performed or supervised by one of us (A.T.) at a single institution.

The patients were randomized into three groups with 20 patients each: Group 1 patients received local 20mL of levobupivacaine 0.25% (Chirocaine™, Abbott) infiltration to the trocar incisions before skin closure. All injections were performed under direct vision into the region of the fascia and preperitoneal tissue at the trocar sites. The infiltration volume was divided equally among the trocar sites. In group 2, 1g paracetamol (Perfalgan™, Bristol MS) was given to the patients intravenously 30 minutes before extubation and 5g paracetamol (Perfalgan™, Bristol MS) was given intravenoulsy in the next 24 postoperative hours. In group 3, lornoxicam (Xefo™, Nycomed) 8mg i.v. was given 30 minutes before extubation and 8mg. lornoxicam i.v. was given in the 24 hours postoperative period. The study drugs were prepared by our anaesthesiologist (A.P.). Randomization was performed by drawing the allocation group code from a sealed envelope.

During the operation, all patients were monitored with electrocardiograph, peripheral oxygen saturation, non-invasive systemic blood pressure, end-tidal carbon dioxide partial pressure. Anaesthetic induction and management were standardized. Haemodynamic and ventilatory parameters were recorded every 5 min. All patients were extubated on the operating table and admitted to the recovery room. Pain was measured by means of a 10-point visual analogue scale (VAS) ranging from 0 (no pain) to 10 (severest pain ever experienced) immediately after admittance to the recovery room (time 0), and at postoperative intervals of 1, 2, 4, 12 and 24 hours. Pain scores were reported as pain at rest. If the patient reported a range for the pain score, i.e 6 to 7, the higher score was recorded. The evaluation of pain scores of our patients was made by one of us (M.B.).

Postoperatively all patients were treated with a patient controlled analgesia (PCA) device (Abbott Life Care PCA Plus Infuser, Abbott La-oratories, Chicago, USA) for pain control using tramadol HCl (Contramal™, Abdi Ibrahim). The PCA technique and the VAS were explained to the patients during the preoperatively. Patients were connected to the PCA device on arrival to recovery room. The PCA solution contained tramadol 2mg/ml. The administration variables were as follows: initial dose 25 – 100mg; incremental dose until achieve VAS score ≤4, tramadol was given at 5 minutes intervals and each time 25mg; PCA device has set: 10mg; lockout interval, 15 min.; 4 hours limit, 150mg; and no basal infusion. The PCA unit was discontinued after 24 hours. The cumulative tramadol consumption was recorded into the PCA device's memory. If the VAS was superior to 4, pethidine (Aldolan™, Liba) 50mg i.m. was administered, maximal every 4 hours. The amount of postoperative parenteral pethidine (Aldolan™, Liba) medication was recorded.

In the present study, we also assessed quality of recovery using Quality of Recovery Score Scale (5). This score was evaluated at postoperative 24th hours by our anaesthesiologist (A.P.).

Patients, the data collector, all patient caregivers and those who assessed postoperative VAS score and Quality of Recovery Scale score were blinded to treatment assignment.

### Exclusion criteria

The patients who could not be able to ascertain the scale, with previous abdominal surgery history, alcohol and/or drug abuse, allergic reaction history to local anaesthetic agents, history of gastric ulcer, bleeding diathesis, abnormal liver and renal functional tests were not included in the study.

### Statistical analysis

Statistical analysis was performed by Statistical Packet for Social Sciences (SPSS, Chicago, Il, USA) version 13.0 for Windows. The data were analyzed and compared using the repeated measure variance analysis, one-way ANOVA test, chi-square test and Fisher's exact test. A p value less than 0.05 was considered significant.

## RESULTS

The demographic, preoperative and postoperative parameters were similar among the groups (Table-1). Postoperative pain severity of the patients significantly decreased in each group at recovery room (time 0) and postoperative 1st, 2nd, 4th, 12th and 24th hour (repeated measured variance analysis, p < 0.001). However, the reduction of postoperative VAS scores was in all times similar among the groups (repeated measured variance analysis, test, p = 0.466) (Table-2).

**Table 1:** Patient characteristics, operative and postoperative details.

Parameters	Group 1 (n=20)	Group 2 (n=20)	Group 3 (n=20)	p value
Age (years)	47.4±10.5	49.4±13.2	48.3±15.9	0.808*
Gender (Male/Female)	11/9	11/9	10/10	0.935**
Body mass index (kg/m^2^)	27	28	28	0.536*
Duration of surgery (min)	128.8±68	135.4±51	134.6±57.6	0.564*
Type of surgery (RCD/SN/A/RN)	2/4/4/10	2/2/4/12	2/3/4/11	0.993***
Lenght of stay (d)	2.2±1.2	1.9±1.8	2±1.4	0.426*

* One way-ANOVA test;

** chi-square test;

*** Fisher's exact test.

Variables indicated as mean ± standard deviation.

**RCD** = Renal cyst decortication; **SN** = Simple nephrectomy; **A** = Adrenalectomy; **RN** = Radical nephrectomy

**Table 2:** Mean postoperative visual analogue scale scores.

POSTOPERATIVE TIME	Group 1 (n = 20)	Group 2 (n = 20)	Group 3 (n = 20)
0 min	7.4 (0-10)	6.8 (0-9)	7.0 (0-10)
1 hour	4.3 (3-7)	3.6 (1-7)	4.0 (1-8)
2 hours	3.9 (2-6)	2.7 (0-6)	3.2 (0-8)
4 hours	3.1 (0-8)	2.7 (0-6)	2.6 (0-7)
12 hours	2.4 (0-6)	2.1 (0-5)	2.0 (0-5)
24 hours	2.0 (0-4)	2.1 (0-5)	1.9 (0-3)
p value*	< 0.001	< 0.001	< 0.001

* Repeated measured variance analysis.

Minimum and maximum values are indicated in parenthesis.

The calculated cumulative tramadol consumptions at 24th hours per kilogram were 370.6 ± 121.6mg, 220.9 ± 92.5mg and 240.7 ± 110.4mg in groups 1, 2 and 3, respectively. We found significant differences in relation to tramadol consumption between groups 1 and 2 and groups 1 and 3 (one-way ANOVA test, p < 0.001). Nevertheless, we did not find significant differences between groups 2 and 3 (one-way ANOVA test, p > 0.05). The percentage of patients in need of pethidine were 5 (33%), 2 (13%), and 2 (13%) in groups 1, 2 and 3, respectively. The average dose of pethidine administered was significantly lower in groups 2 and 3 compared with group 1 (group 1: 145 mg, group 2: 100mg, group 3: 100mg), (one-way ANOVA test, p = 0.024).

In the postoperative period, 3 patients had nausea and no patient vomited in group 1. In group 2, 5 patients had nausea and 1 patient vomited. In group 3, 6 patients had nausea and 1 patient vomited (one-way ANOVA test, p = 0.612). There were no undesirable events in any group.

The Quality of Recovery Scale scores did not significantly different among the groups (Table-3).

**Table 3:** Mean Quality of Recovery Scale scores.

Parameter	Group 1 (n = 20)	Group 2 (n = 20)	Group 3 (n = 20)	p value*
Quality of Recovery Scale score	14.4 (13-16)	15.5 (13-17)	15.9 (13-18)	0.385

* One way-ANOVA test.

Minimum and maximum values are indicated in parenthesis.

## DISCUSSION

One of the major benefits of laparoscopic techniques compared with standard open surgery is the reduction of postoperative pain. Nevertheless, it is recognized that the skin and fascial incisions through which laparoscopic trocars are inserted can still lead to significant degree of pain in postoperative period (6). Effective analgesic therapy is an important component of postoperative follow-up. For this reason, opioids are the most preferable analgesic agents during early postoperative period after laparoscopy. However, opioids could cause some serious adverse effects such as over sedation, depression of respiration and gastrointestinal motility, nausea, urticaria and vomiting (7, 8). To continue to improve the patient experience with laparoscopy and to minimize the opioid analgesia required after surgery, some additive and synergic drugs such local anaesthesia infiltration through incisional trocar sites, parenteral non-steroidal anti-inflammatory drugs (NSAID), parenteral paracetamol, and intraperitoneal local anaesthesia instillation are used in pain management after laparoscopic surgery (9).

Intravenous paracetamol is a fast and effective drug that could support the effects of opioids with fewer side effects such as NSAID which cause gastrointestinal bleeding and renal insufficiency. The analgesic action mechanism of paracetamol has not been well defined yet there are some publications about its anti-nociceptive effects. The analgesic effects could be correlated with ratio and amount of the active metabolite of paracetamol (9–11).

Lornoxicam is a NSAID belonging to the enolic acid. It inhibits cyclooxygenase 1 and 2 enzymes (12). In the treatment of postoperative pain, lornoxicam has been shown to be as effective as pethidine (12), morphine (13), and tramadol (14, 15). Moreover, in the postoperative pain management, lornoxicam has been well tolerated. Its short plasma half-life (approximately 4 hours) may provide advantages over other NSAIDs which have been linked to a high incidence of adverse effects (12–15).

The ease of use and safety of local anaesthetics is well recognized. The main advantage of local anaesthetic agents is that they do not have the adverse effects of systemically administered opioids. Local anaesthetic agents are commonly administered in abdominal surgery by skin infiltration or epidural administration, blocking somatic afferents and providing significant benefits in reducing postoperative pain and improving recovery (16). Levobupivacaine which is a long-acting local anaesthetic agent reversibly blocks the transmission of action potential in sensory, motor and symphatetic nervous fibers by inhibiting the passage of sodium through voltage-sensitive ion channels in the neuronal membrane (17). In some laparoscopic procedures, subcutaneous infiltration and intraperitoneal instillation of levobupivacaine have been the subject of several published studies (18–20).

Previous researches have mainly examined the efficacy of paracetamol, NSAIDs, and local anesthetic agents in reducing pain after laparoscopic cholecystectomy (LC). However, little attention has been paid to understanding the effects of these agents following urologic laparoscopic surgery. In the literature, some studies assessed the benefit of paracetamol treatment after LC. In a recent study, the authors studied the efficacy of paracetamol or valdecoxib after LC. The authors reported that paracetamol is as effective as valdecoxib for pain after LC. The effect of dexamethasone was similar in paracetamol and valdecoxib patients (21). In another recent study, the effect of intravenous paracetamol treatment on early postoperative analgesia and recovery characteristics after LC have been assessed (22). In this study, 40 patients underwent LC and were randomly divided into two equal groups. In the first group, 1g intravenous paracetamol was given to the patients after intubation 15 minutes before the surgery started. One-hundred ml saline was infused intravenously for the control group in 15 minutes. According to the results, verbal and visual pain scores of the paracetamol group were significantly lower than the other group. Furthermore, first morphine requirement and total administered morphine dose and the length of stay in the recovery room were significantly decreased in paracetamol group. The authors concluded that beside its effective analgesic properties, paracetamol administration during per-operative period supports effective and faster recovery. These two studies mentioned above show that paracetamol seems to be a good option for pain control after laparoscopic surgery.

Several studies have documented lornoxicam and other NSAIDs efficacy for the management of postoperative pain after laparoscopic surgery. In a study by Papadima and associates, they compared the efficacy of lornoxicam vs. parecoxib (cyclooxygenase-2 enzyme inhibitor) for pain control after LC (23). In this study, 76 patients were randomized to receive before induction parecoxib 40mg i.v., lornoxicam 8mg i.v. or placebo. They stated that parecoxib and lornoxicam were equianalgesic and both were more efficacious than placebo for pain control after LC. In another study, the authors reported that pre-operatively administered lornoxicam 16mg i.v. significantly prolonged the first morphine demand time and reduced postoperative morphine consumption when compared with tenoxicam 40mg i.v. after LC (24).

Instilling long acting local anaesthesia into the laparoscopic trocar incisional sites has been supported for LC, laparoscopic appendectomy, diagnostic gynecologic laparoscopy and laparoscopic renal/adrenal surgery. In a study by Herkanwal et al., a total of 72 patients underwent transperitoneal laparoscopic renal and adrenal surgery and were randomly assigned to the treatment (0.5% bupivacaine) or placebo (0.9% saline) arms (25). Trocar and hand assisted port sites were infiltrated at the end of the procedure. The authors concluded that at the outset of transperitoneal laparoscopic renal and adrenal surgery, trocar site and other incision infiltration with long acting local anaesthesia decreases postoperative parenteral opioid requirements compared with placebo controls. In another study, 57 patients scheduled for LC were randomly assigned to receive local infiltration with 0.9% saline solution (n = 18), ropivacaine 1% (n = 20) and levobupivacaine 0.5% (n = 19) (18). The local anaesthetic was administered prior to trocar placement, using the same technique and delivering the same volume (20ml) for all three groups. The authors stated that local tissue infiltration with levobupivacaine was more effective than ropivacaine in reducing the postoperative pain associated with LC. Louizos and co-workers reported that pre-incisional levobupivacaine 0.25% infiltration significantly reduced postoperative pain after LC (19). Conversely, Savaris et al. reported that the use of pre-incisional local injection of levobupivacaine 0.5% in the trocar sites was not superior to placebo to reduce pain after laparoscopic tubal ligation (26). A most recent review reported that preemptive incisional local anaesthetic was superior to placebo with regards to postoperative pain control. The authors stated that timing of administration (prior to actual skin incision or late infiltration) was not significant for impact of local analgesia (27).

To our knowledge no comparison of the efficacy of intravenous paracetamol, intravenous lornoxicam and levobupivacaine infiltration into the trocar sites for pain control after transperitoneal laparoscopic renal and adrenal surgery has been published. Our data suggest that postoperative pain degree of the patients significantly decreased in the groups. This result has not surprised us and it was consistent with the literature. In the present study, the reduction of postoperative VAS scores was similar among the groups. Because all patients were allowed to receive tramadol via PCA device for autonomous pain control, it was not expected that pain scores would vary among the groups. We found the levobupivacaine treated patients required significantly more intravenous tramadol via PCA device and additional pethidine administration when compared with paracetamol and lornoxicam groups. The amounts of tramadol and pethidine consumptions did not differ between paracetamol and lornoxicam groups. Although the study did not address the ideal timing of trocar site infiltration, late infiltration of levobupivacaine might be associated with high amounts of tramadol consumption and additional pethidine requirements in our patients.

## CONCLUSIONS

In the light of results, intravenously paracetamol and lornoxicam administration seems equianalgesic and both are more efficacious than levobupivacaine infiltration without serious adverse effects for pain control after transperitoneal laparoscopic renal and adrenal surgery. Our results show that both parenteral paracetamol and parenteral lornoxicam therapies could be chosen as options in patient treated with upper urinary tract transperitoneal laparoscopic surgery. We believe that this issue should be studied further with more patients.
